# Space-Time Cluster’s Detection and Geographical Weighted Regression Analysis of COVID-19 Mortality on Texas Counties

**DOI:** 10.3390/ijerph18115541

**Published:** 2021-05-22

**Authors:** Jinting Zhang, Xiu Wu, T. Edwin Chow

**Affiliations:** 1 School of Resource and Environmental Science, Wuhan University, Wuhan 430079, China; whuzjt@whu.edu.cn; 2 Department of Geography, Texas State University, San Marcos, TX 78666, USA; chow@txstate.edu

**Keywords:** geographical weighted regression, space-time cluster’s detection, COVID-19, mortality

## Abstract

As COVID-19 run rampant in high-density housing sites, it is important to use real-time data in tracking the virus mobility. Emerging cluster detection analysis is a precise way of blunting the spread of COVID-19 as quickly as possible and save lives. To track compliable mobility of COVID-19 on a spatial-temporal scale, this research appropriately analyzed the disparities between spatial-temporal clusters, expectation maximization clustering (EM), and hierarchical clustering (HC) analysis on Texas county-level. Then, based on the outcome of clustering analysis, the sensitive counties are Cottle, Stonewall, Bexar, Tarrant, Dallas, Harris, Jim hogg, and Real, corresponding to Southeast Texas analysis in Geographically Weighted Regression (GWR) modeling. The sensitive period took place in the last two quarters in 2020 and the first quarter in 2021. We explored PostSQL application to portray tracking Covid-19 trajectory. We captured 14 social, economic, and environmental impact’s indices to perform principal component analysis (PCA) to reduce dimensionality and minimize multicollinearity. By using the PCA, we extracted five factors related to mortality of COVID-19, involved population and hospitalization, adult population, natural supply, economic condition, air quality or medical care. We established the GWR model to seek the sensitive factors. The result shows that adult population, economic condition, air quality, and medical care are the sensitive factors. Those factors also triggered high increase of COVID-19 mortality. This research provides geographical understanding and solution of controlling COVID-19, reference of implementing geographically targeted ways to track virus mobility, and satisfy for the need of emergency operations plan (EOP).

## 1. Introduction

The coronavirus disease 2019 (COVID-19), as a global disaster, inhibited social-economic development worldwide in 2020. It has threatened the loss of human life, public health, safety, and disruption of face-to-face communication due to intangible, clinical severity of the infection, and fatal symptoms [1]. By 11 March 2021, 2.62 million lost their lives around the world, accounting for 15% of World War One fatality. A pervasive sense of quarantine fatigue and panic attacks of getting infected are challenging human resilience [2,3]. COVID-19 is one of the extreme diseases as incurable and universally fatal, killing 25–50% of patients [4]. In particular, the COVID-19 pandemic in the US was exposed to mass dislocation, directly accelerating the decline and failure of public health. With around 30 million diagnosed cases and over 540,000 deaths as of mid-January 2020, a disproportionate impact on COVID-19 was produced. About 40% of cases should have been averted with international cooperation of medical care [4]. In addition, age-specific mortality rates in the United States had remained corresponding to the weighted average of G7 nations [4]. 

Texas is the second-largest state in the United States and has one-tenth of the aging people. Despite unremitting Texas Executive Orders (TEO) and Public Health Disaster Declarations (PHDD) were made, the Texas government maintained economic openness. The first COVID-19 case in the United States was confirmed on 19 January 2020, in Washington State [5], whereas the first case was announced by The Texas Department of State Health Services on 4 March in Fort Bend County. As of 28 February 2021, Texas surpasses 2,300,000 total COVID-19 cases and 372,086 deaths cases. As United States has gone through several waves of epidemic cycles, Texas has undergone all five stages of COVID-19 risk-based guidelines. Texas disease surveillance and response systems have disclosed the vulnerability to deal with the global pandemic, which underlines the requirement to establish global scheme, regulation, and collaboration [6]. A silver lining is that the pandemic provides a unique and empirical opportunity to observe a large-scale and prolonged episode of public health emergency. Accordingly, it is imperative to understand the spatial-temporal clusters of COVID-19 mortality and explore its relationships with environmental and social-economic factors.

A popular statistical tool to look into that relationship is space-time scan statistic, which is widely used to quantify cluster strength and statistical significance [7]. Epidemic surveillance and spatiotemporal trending analysis can provide unique insights for decision-makers to be aware of potential uptakes and adopt proactive public health measures to mitigate the risk and minimize COVID-19 infection. Detecting patterns of COVID-19 confirmed cases and mortality in the United States are well documented to formulate interventions, targeted rapid testing, and resource allocation [8,9,10]. However, the usefulness of space-time analysis depends on the data quality (e.g., accuracy, spatial resolution, temporal currency, completeness, etc.), which are somewhat limited at the early stages of pandemic. Besides, Desjardins mentioned deaths could be conducted, but not incorporated in the research scope. Those spaces are filled in our study. The distribution of the COVID-19 pandemic is well represented by Geographical Information Systems (GIS) spatial analysis with the multidimensional social, economic, and health consequences, exposing to geographical inequity and a long-term impact on global health accurately [11,12,13]. GIS-driven spatial analysis can facilitate the combination between health data and characteristic of spatial attributes. Descriptive modeling research that took advantage of those strength has deeply exposed the spatial-temporal associations of COVID-19 with socioeconomic and environmental characteristics [14,15]. However, as far as an engaging empirical study, it is important to select variables that reveal the degree of social vulnerability [16,17,18]. 

Spatial-temporal analysis of COVID-19 is crucial to understanding the spread of COVID-19 and explore appropriate community containment strategies, which are fundamental public health measures used to control the spread of communicable diseases, including isolation and quarantine. This paper focuses on the county level within a state to eliminate the possibility of policy divergences between states, since existing research spatial-statistically calculated county-level data, but not temporal lag disparity of county-level [19,20,21,22,23]. Due to varying social vulnerability associated with different population demographics, such as age, gender, and race/ethnicity, some population groups are more vulnerable to the threat of COVID-19. A few variables are presented in the previous modeling [24,25,26,27,28,29], albeit population mobility, age, race were significant factors [30,31,32,33,34,35,36]. As a respiratory disease, air pollution indices like PM2.5 and air quality index (AQI) are highly related to COVID-19. Despite air quality, Qian contends, is viewed as a robust interaction with COVID-19 [37], AQI and PM2.5 have not been explored in previous spatial-temporal models, only added as impact factors on the environmental list [38,39,40,41,42]. 

The research purposes are of two folds—first, to identify any emerging space-time clusters of COVID-19, and second, to examine any significant factors related to mortality. By exploring the spatiotemporal clusters based on a more comprehensive set of data over a year-long period, this research examines the correlation between COVID-19 mortality rate and social-economic, environmental factors with GWR analysis. It aims to identify sensitive indicators to assist the formulation of targeted intervention suitable for vulnerable populations and break the chains of transmission. Hence, this research is expected to provide references for preventing and controlling COVID-19 and related infectious diseases, evidence for disease surveillance, and response systems to facilitate the appropriate uptake and reuse of geographical data, to contribute to safeguarding Texas public health. Our long-term goal is to improve and strengthen health seamless connection and surveillance system by timely dynamic monitor mechanism.

## 2. Materials and Methods

### 2.1. Data 

COVID-19 mortality is the subject of observation in the space-time scan statistic. The COVID-19 mortality rate as the dependent variable is acquired from the Centers for Disease Control and Prevention (CDC), COVID-19 fatality data based on death certificates. A fatality is counted as a COVID-19 fatality when the medical certifier attests to the death certificate that COVID-19 is a cause of death. Mortality rate is equal to fatalities divide by cumulative cases. Hospitalization (i.e., total hospital bed, bed per capitalPC) from The Texas Department of State Health Services (DSHS) is reported daily by hospitals through eight Hospital Preparedness Program providers that coordinate health care system preparedness and response activities in Texas. The data were collected from 4 March 2020 to 1 March 2021 as explanatory variables. Demographic data, such as race, age group, gender, population density, are acquired from the 2020 U.S Census Bureau. Economic data (e.g., annual income) in 2020 are obtained from the Texas Association of Counties, the statistical period is 2020. Environmental data are interpolated from limited samples collected by the United States Environmental Protection Agency (i.e., AQI, PM2.5) and National Weather Service (i.e., temperature, precipitation) during 1 March 2020–28 February 2021. All variables are in Table 1.

### 2.2. Study Framework

**Figure 1:**
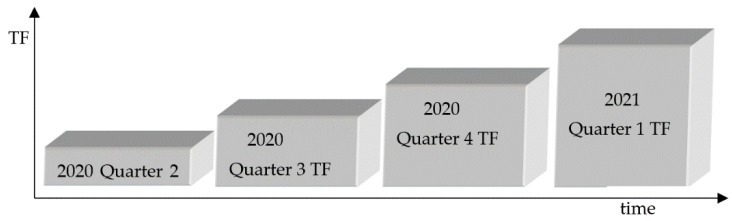
Temporal-study framework.

From a temporal study framework perspective, the study period was classified into four boxes based on the number of fatalities (TF) per quarter. Quarterly statistical data are based on environmental and socio-economic indices at the end of each quarter in response to COVID-19 fatalities at that time. The temporal-study framework in Figure 1.

**Figure 2:**
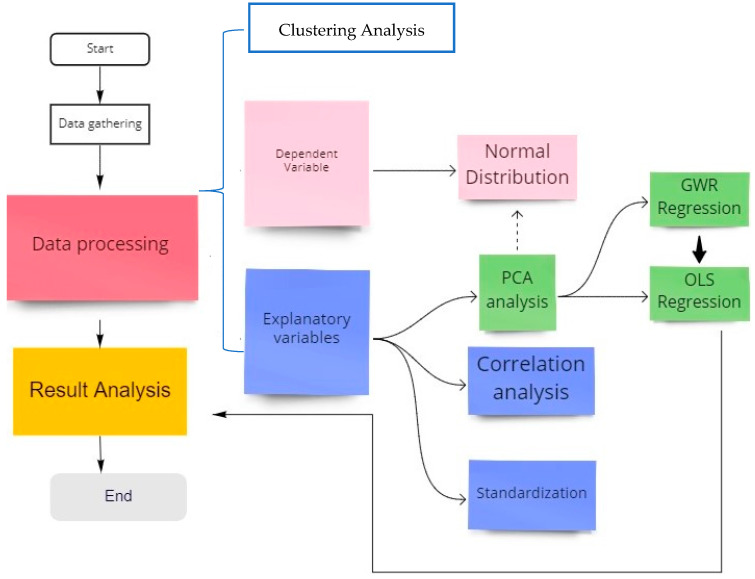
Spatial study framework (PCA = Principal Component Analysis; GWR = Geographical Weighted Regression; OLS = Ordinary Least Square).

For the spatial study, we explore the inter-correlations among independent variables before building the GWR models. Since dependent variables must meet the assumption of a normal distribution, we have to describe their statistical characteristics and spatial autocorrelation analysis. To minimize any multicollinearity, all explanatory variables are standardized and examined by principal component analysis into composite factors. After that, we try to model simple ordinary lease square (OLS) and geographically weighted regression between variables. Finally, via model comparisons, we pay more attention to their differences in spatial heterogeneity and analyze how did it happen, as shown in Figure 2.

### 2.3. Space-Time Scan Statistics

In Kulldorff’s scan statistic method, the first step is to determine a congruous probability model of data, then compute the likelihood ratio test statistic λ(z) for each scan window z. After that, we identify primary cluster candidates with the maximum λ(z), a Monte Carlo hypothesis procedure tests the statistical significance and obtains a *p*-value [43]. On the one hand, Kulldorff’s method tests the null hypothesis H_0_ (constant probability for all areas) and the alternative hypotheses H_1_ (the specific area z has a larger probability than outside areas) using a Poisson model [7]. For a given region z, the likelihood function based on the Bernoulli model can be expressed using Equation (1):

(1) L(z)=Lp>qsup(z,p,q)=(p)nz×(1−p)μ(z)−nz×qnG−nZ×(1−q)(μ(G)−μ(z))−(nG−nZ)

where, *μ(G)* and *μ(Z)* are the total population of the study area and population in region *Z*; *nG* and *nZ* are the total numbers of observed cases in the study area and in region *Z*; *p* is the probability that an incident falls in region *Z*, and *q* is the probability that an incident falls in the rest of the study area. The likelihood of observing *n (Z)* in region *z* is given by the function shown below:

(2) L(z)={Lp>qsup(z,p,q)=p^nZ×(1−p^)μ(Z)−nZ×q^nG−nZ×(1−q^)(μ(G)−μ(z))−(nG−nZ) if p^>q^p^0nG×(1−p^0)μ(G)−nG 

where, =0p^nGμ(G), $\hat{p}=\frac{nZ}{\mu \left(z\right)}$, and $\hat{q}=\frac{nG-nZ}{\mu \left(G\right)-\mu \left(z\right)}$. The expected likelihood function has the form as given in Equation (3):

(3) L0=Lp=qsup(Z,p,q)=(nGμ(G))nG×(μ(G)−nGμ(G))μ(G)−nG 

Therefore the likelihood ratio λ(z) can be obtained as the quotient by dividing the observed likelihood by expected likelihood:

(4) (z)={L(z)L0=Lp>qsup(z,p,q)Lp=qsup(z,p,q) if p^>q^1

Kulldorff (1997) also gave the formula to calculate the likelihood ratio based on the Poisson model as shown below [7]:

(5) $$\left(z\right)=\{\begin{array}{c} \frac{L\left(z\right)}{L_{0}}=\frac{{\left(\frac{nZ}{\mu \left(Z\right)}\right)}^{nZ}\times {\left(\frac{nG-nZ}{\mu \left(G\right)-\mu \left(Z\right)}\right)}^{nG-nZ}}{{\left(\frac{nG}{\mu \left(G\right)}\right)}^{nG}}\;if\;\hat{p}>\hat{q}\\ 1 \end{array}$$

On the other hand, Kulldorff’s method tests the statistical significance of the detected clusters. According to the Monte Carlo simulation, the *p*-value is used to assess the statistical significance of the detected clusters. The Monte Carlo simulation, proposed by Dwass in 1957 [44], Turnbull et al. took advantage of it at their cluster detection tests [45]. In a Monte Carlo simulation, a large number of random replications can be generated under a chosen distribution model, conditioned by the simulated case number as real data. In this study, the real population is used to calculate each area in the Monte Carlo replication. The disease occurrence in each area is gathered from a non-homogeneous Poisson distribution with mean *μ(z) nG μ(G)*. The likelihood ratio is calculated by using the replica data and the real data. Each simulated dataset has a maximum likelihood ratio and *p*-values. The smaller *p*-value and the bigger likelihood ratio generates more likely cluster. The problematical propositions are reliant on scan windows with predefined shapes [46].

### 2.4. Expectation-Maximization Clustering and Hierarchical Clustering Analysis

Two common clustering methods are partitioning clustering and hierarchical clustering. Partitioning cluster analysis pinpoints clusters with similar instances after a set of unlabeled data are given. For example, expectation-maximization algorithm clustering (EM) conducts maximum likelihood estimation for samples in a mixture model. EM utilized probability of cluster membership rather than a distance metric, and samples are not assigned to one cluster but partially to distribution. It is common in chronic diseases clustering detection such as diabetes patients, that tend to form groups that are either intersection or undependable shapes [47]. Hierarchical clustering is a method of automatically seeking a hierarchy of clusters, which is a general application of DNA cluster detections. It includes agglomerative clustering (i.e., bottom-up approach) and divisive clustering (i.e., top-down approach). Both EM and hierarchical clustering belong to machine learning analysis. They do not dependent on the predefined window and arbitrary patterns to detect clusters.

### 2.5. Selection of Explanatory Variables

To reduce the dimensionality of the dataset down to fewer explanatory variables, principal component analysis (PCA) is one of the common techniques to minimize multilinearity without losing the attribution of variables. PCA could maintain interpretability while minimizing information loss. It does so by creating new independent factors or components that successively maximize variance. In the PCA procedure, a set of possibly correlated variables is transformed into a set of linearly uncorrelated variables using the orthogonal transformation. The number of factors extracted from PCA is less than or equal to the number of previous possibly correlated variables [48].

### 2.6. Model Selection

Owing to spatial dependence of COVID-19 spreading, the purpose of modeling (Mortality Rate) MR is to figure out the external triggers that took place readily. Statistical modelling is a good way to be considered to make predictions about the real world via sample data. For instance, the ordinary least square (OLS) is a traditional method for estimating a linear regression between dependent and independent variables. OLS assumptions involve the disturbances that have zero mean and constant variance, in addition to no correlation among explanatory variables [49]. However, multicollinearity in OLS can cause bias of the model, inflate model performance, and influence the reliability of the outcome. Then, to mitigate multicollinearity, stepwise regression (SR) is one of the common approaches to be considered. SR is an automatic variable selection procedure that selects the most related candidate(s) among a pool of explanatory variables iteratively. Forward selection begins with no variables in the model, examining each additive variable with a chosen model-fit criterion until none of the remaining variables improve the model to a statistically significant extent [50]. In this study, SR is disregarded due to biased R-square or coefficient [51]. The GWR modeling is initially taken into account for the geographical disproportion of the number of deaths [52]. More importantly, compared to OLS models, GWR models are local linear regression models. They embrace the calculation of a parameter estimate of variations over space in the link between independent and dependent variables [53,54].

### 2.7. GWR

The GWR procedure is founded upon two conditions. First, similarities between more adjacent geographical entities exist based on the first law of geography [55]. Second, there are disproportionate distribution of explanatory variables (e.g., socioeconomic factors) in different regions, due to spatial autocorrelation and spatial heterogeneity. Based on Foster’s spatial varying parameter regression, a Geographically Weighted Regression model (GWR) is localized through weighting each observation in the dataset [54]. As pointed out by Fotheringham, local smooth processing was used to address the spatial heterogeneity. Under the consideration of spatial disparity, geographic coordinates and core functions are utilized to carry out local regression estimation on adjacent individuals of each group. The equation of the GWR fitted model is as follows [54].

yi = β_0_(u_i_, v_i_) + ∑_k_β_k_(u_i_, v_i_)x_k,i_ + ε_i_ (6)

where i denotes the individual sample; (u_i_, v_i_) is the coordinates of sample i; β_k_(u_i_, v_i_) is the k_th_ regression parameter of sample i; y_i_ is the dependent variable of sample i, x_k_, i is the k_th_ independent variable for the sample i, ε_i_ is random error term which obeys normal distribution when the variance is a constant, thus the parameter estimation value of sample i is given by:

(7) $$\hat{\beta }(u_{i},v_{i})={\left(X^{T}W(u_{i},v_{i})X\right)}^{-1}X^{T}W(u_{i},v_{i})y$$

where W is the spatial weight matrix, whose selection and setting are the core issues of GWR regression. The calculation of GWR coefficients consists of two major steps—first by selecting a proper kernel function to express the spatial relationship between the observed units. Specifically, four major kernel functions are used in the existing research, namely fixed Gaussian, fixed Bi-square, adaptive Bi-square, and adaptive Gaussian. Since the merits of a kernel function play a direct and decisive role in obtaining the most accurate possible regression parameter estimation of spatial heterogeneity, after careful analysis and comparison, fixed Gaussian was chosen as the kernel function in the paper, which is expressed as,

(8) $$w_{\mathrm{ij}}=\exp\left(-d_{\mathrm{ij}}^{2}/\theta^{2}\right)\;$$

where w_ij_ represents the distance weight from sample i to sample j; d_ij_ is the Euclidean distance between sample I and sample j; θ is the bandwidth, which determines the speed at which the spatial weight attenuates with distance. The second step of spatial weight matrix calculation is the selection of optimal bandwidth which could contribute to a higher fitting degree. According to the GWR4.09 User Manual [55], bandwidth selection criteria include AIC (Akaike Information Criterion), AICc (small sample bias-corrected AIC), BIC, and CV (Cross Validation).

## 3. Results

### 3.1. Space-Time Scan Statistics

**Figure 3:**
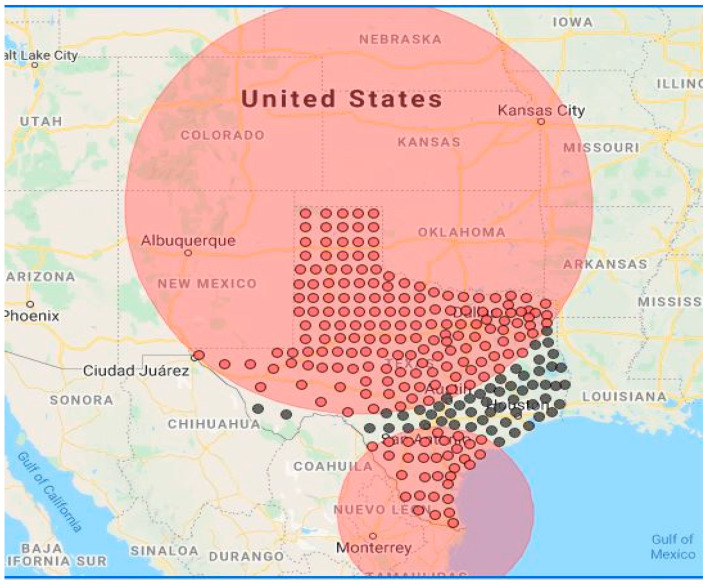
Space-time clusters distribution map. (red points mean TX counties inside clusters, black points mean TX counties outside clusters.)

By using SaTCan software (Harvard Medical School and Harvard Pilgrim Health Care Institute, 133 Brookline Avenue, 6th Floor, Boston, MA 02215, USA), two significant space-time clusters of COVID-19 were detected at 0.05 level (Figure 3; Table 2). The bigger cluster incorporates 172 counties of 13,085,347 population and 12,761 new cases, covering the northern and western Texas. During the period of 6 November 2020–5 February 2021, this cluster observed COVID-19 cases that were 2.48 times more than expected cases. The second cluster centers around East Texas and involves 27 counties with 26,217,888 population and 3635 new cases during 6 July 2020–5 September 2020. This eastern cluster has an observed/expected ratio of 5.23 times. It is noted, however, that this eastern cluster took place during the earlier stage of the pandemic when the COVID-19 cases had just started spreading in Texas and hence the expected cases were lower than the northern cluster. Among the 254 counties in Texas, these two clusters occupied 199 counties. The spatial extent of these clusters is too large to guide precise tracking of COVID-19 mortality.

**Figure 4:**
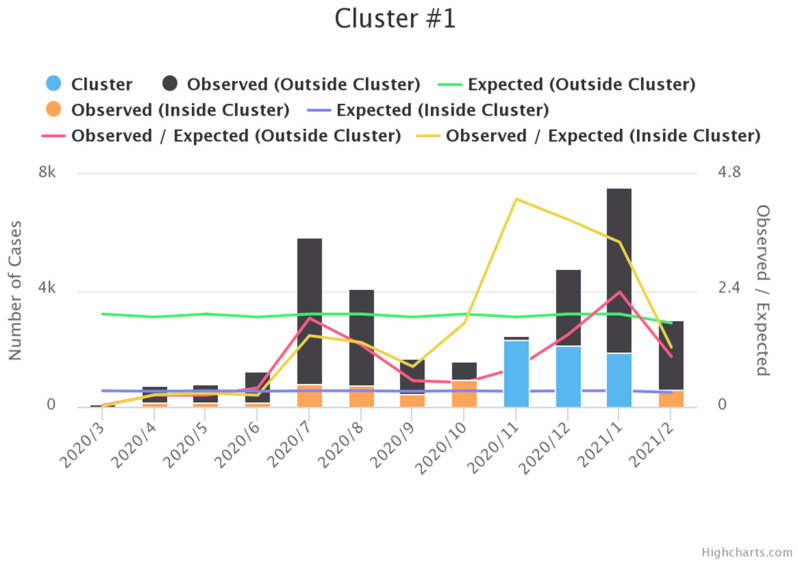
Temporal trend. (We used Highchart.com to generate above chart and accessed on 20 March 2021).

Focusing on the temporal trend, November 2020 is the most serious month in the 3 months space-time cluster in the northern and western Texas (Figure 4). According to the above Figure 4, the highest month of the proportion of observed/ expected cases is shown in November 2020. Hence, the cluster period is confirmed in the last two quarters of 2020 and the first quarter of 2021, and the cluster’s locations covered 199 counties, which is the key of the following GWR analysis.

### 3.2. EM Clustering and HC Clustering

Based on mortality rate alone, the EM algorithm identified seven clusters in the third quarter that are not significant (Table 3). In the last quarter, eleven clusters are produced, including seven significant clusters and four insignificant clusters. The maximum log likelihood is −86.34. In HC clustering, the cases are classified as cluster 0 and cluster 1.

Cluster 0 means four counties as a group in the third quarter and eight counties as a group in the last quarter, including Cottle, Stonewall, Bexar, Tarrant, Dallas, Harris, Jim hogg, and Real. Incorrectly clustered instance are 251 counties in the third quarter and 247 counties in the last quarter. Two clustering methods selected classes to cluster evaluation parameters. They are prior to the previous space-time cluster detection due to narrow county scales.

### 3.3. Normal Distribution

Based on the above analysis, normal distribution was conducted on two clusters in the last two quarters of 2020 and the first quarter of 2021. The request for normal distribution has two conditions. One is uncertain variable that is symmetric about the mean, another is the uncertain variable that is more likely to be in the vicinity of the mean than far away. After the logarithm transformation, MR is qualified.

### 3.4. Correlation

According to Table 4, in the third quarter, MR is positively significant to annual income and the population older than 80, but negatively significant to temperature, precipitation, total hospital beds, population density, total population, black population, and the age groups between 20 and 59. In the fourth quarter of 2020, MR is negatively significant to temperature, precipitation, total hospital beds, population density, total population, annual incomes, and the population between 20 and 59, while it is positively significant to population older than 80. Interestingly, annual income began as positively related to MR but then negatively related to MR.

### 3.5. Factor Analysis

Through PCA, the dataset was examined using Kaiser-Meyer-Olkin (KMO) and Bartlett’s Test of Sphericity. The KMO test compares the correlation statistics to identify if the variables include sufficient differences to extract unique factors. A KMO value of 0.616 for 14 explanatory variables is more than the threshold value of 0.5. The Bartlett’s Test of Sphericity (BTS) value of 0.0 was significant (*p* < 0.001), validating that correlation between variables does exist in the population. Communality is a common variance between 0 and 1, using the remaining variables as factors, was used to determine if any variables should be excluded from the factor analysis (Table 5). A 0.7 threshold is used to determine the significance of explanatory variables.

**Figure 5:**
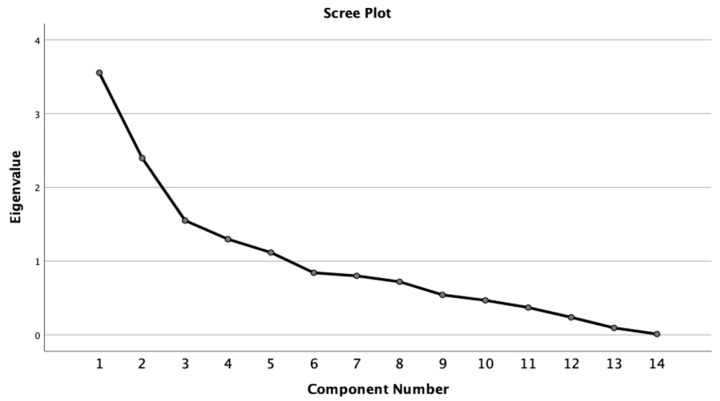
Component factors extracting.

PCA was conducted as the factor analysis method in this paper. Using an eigenvalue threshold greater than 1.0, 5 factors are identified that could explain a cumulative 70.18% of the variance within the data model (Table 6 and Figure 5). A varimax rotation was used to assist in the interpretation of the PCA analysis. The rotated component matrix was examined for variables with a cutoff threshold of 0.7. Table 6 gave us the direct relationship between factors and explanatory variables. The first factor, in three quarters, represents high loading on variables related to CareBeds, Total Population, Population Density, indicating the COVID-19 mortality rate is positively related to hospitalization and total population. That means the metric of population and the index of medical care are two main indicators of COVID-19. Factor 2 in the third quarter of 2020, factor 4 in the first quarter of 2021 and factor 4 in the fourth quarter of 2020 were a composite adult population index related to the population between 20 and 59 and beyond 80. Factor 3 in two quarters of 2020 and factor 2 in the first quarter represent natural supply index, which related to land area and precipitation, indicating keeping social distancing was helpful to mitigate MR. The economic condition indexes include Factor 4 in the third quarter, factor 2 in the fourth quarter, and factor 5 in the first quarter in 2021 through household income and unemployment. Factor 5 in the third quarter of 2020 and factor 3 in the first quarter of 2021 were environmental indexes. Meanwhile, factor 5 in the fourth quarter (i.e., beds per capital), was the medical supply index, positively affecting MR.

### 3.6. Comparison of Composite OLS and Composite GWR Models

The OLS regression examines whether there is a linear relationship between cumulative case and its factors, as well as between death rate and its factors. By the T-test and F-test, all factors were significant. By binning MR by quarter, an iterative approach of GWR is conducted to examine how the spatial relationship between MR and its factors change over time, since MR is clustered and an adaptive kernel in GWR models is adopted. The AICc method chooses the bandwidth that minimizes the AICc value—the AICc is the corrected Akaike Information Criterion (it has a correction for small sample sizes). By comparing the results (Table 7), the AICc value is decreased from 875.23 in the OLS model to 851.54 in the GWR in the third quarter of 2020, whereas R^2^ increased from 0.17 in the OLS model to 0.37 in the GWR models of two quarters. As these two models represent a global and a local approach respectively, the neighbors declined from 254 neighbors in the OLS models to 128 neighbors in the GWR models. In Q4 2020, the same trend of AICc decrease is observed from 665.44 in the OLS model to 653.85 in the GWR, and R^2^ increased from 0.10 in the OLS model to 0.20 in the GWR model. For three times, the GWR model enjoyed higher predictive power than OLS and is hence superior. Despite the GWR model remained moderately weak in modeling MR, the models are significant.

### 3.7. GWR Result Analysis

#### 3.7.1. Spatial Change of MR Factors

**Figure 6:**
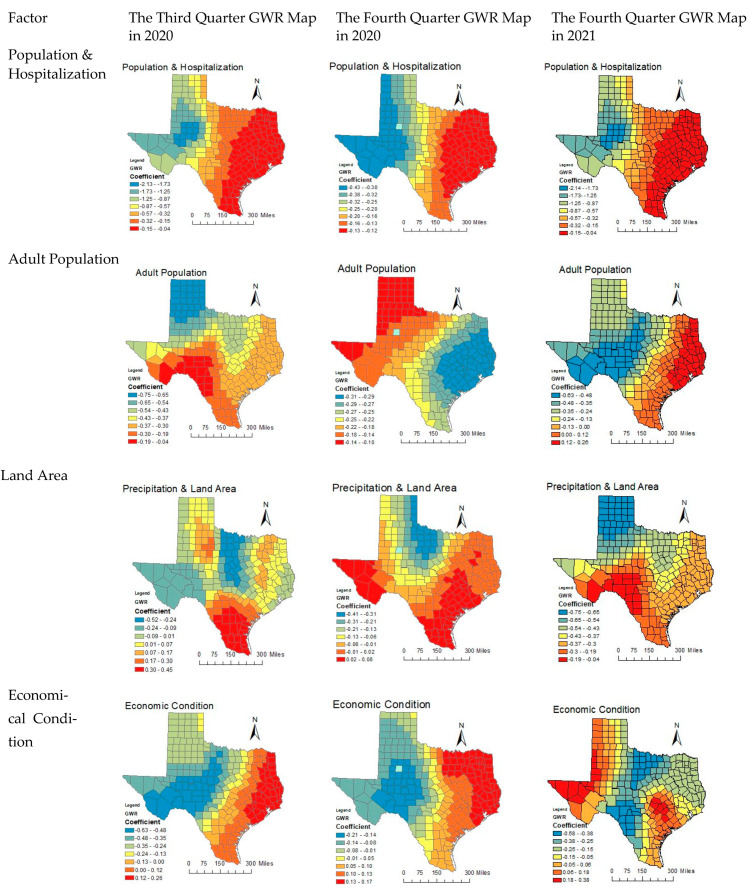

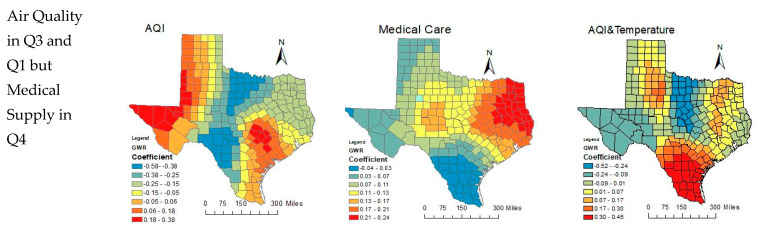
Spatial-temporal GWR map.

Based on existing research, COVID-19 quarterly GWR models are also implemented in the research area [55,56]. Figure 6 incorporates Texas spatiotemporal distribution maps based on five factors in terms of five aspects in three quarters. 

In the third quarter of 2020, Factor 1 among 5 factors has the dominant effect on MR because the maximum range of coefficient is −0.15 to 0.04. It is the lowest impact in Central Texas thanks to the coefficient range of −2.14 to −1.73, implying the hospitalization capacity has not been stressed beyond full capacity. Accordingly, when looking at Factor 1 in the third quarter, all Texas counties were in the negative range which was good. For Factor 2, a high score reflects more population in 20–59 and population less than 80. A negative relationship with MR indicates lower mortality in younger population (but also higher mortality in elderly population). This negative relationship was the strongest in northern TX but weakest in western TX. In addition, the negative values do not mean smallest impact, just the way the relationship is. Interestingly, the progression was south-north oriented in the third quarter but east-west oriented in the fourth quarter. Factor 3 is a natural supply index, having remarkable spatial disparity for its coefficient range −0.52 to −0.24 to range 0.3 to 0.45. In Central Texas, the land area is little driven COVID-19 MR, but it reversely works on South Texas. That indicates spatial distancing is more available for South Texas than Central Texas. Factor 4 is an economic composite index with coefficient from range −0.63 to −0.48 to range 0.12 to 0.26. This is a “bad” economy factor where PCI is negative and UEM is positive. Western TX has negative coefficients meaning bad economy did not result in higher MR, but eastern TX did have positive coefficients which indicates poorer population suffered first. Factor 5 is the air quality index that coefficient is from range −0.58 to −0.38 to range 0.18 to 0.38. AQI is higher with poor air quality. If air quality affects MR of COVID-19, it should have a positive relationship (i.e., the worse the air quality, the higher MR). Hence, a negative relationship means air quality did not matter (regardless of the AQI was good/bad in that area), but there was a positive relationship in West and Central/East TX (near Harris County) when COVID-19 emerged in the third quarter.

In the fourth quarter of 2020, Factor 1 among the 5 factors does not have the dominant effect on MR without the range of maximum coefficient which is −0.43 to −0.12. That means the hospitalization capacity has not been stressed beyond full capacity. Factor 2 is an economic composite index whose coefficient is from range −0.21 to −0.14 to range 0.13–0.17. The Central TX became the divide with neutral relationship in this factor, but western TX remained negative but eastern TX became positive. Factor 3 is a natural supply index with the coefficient from the range −0.41–0.31 to range 0.02–0.08. In northern Texas, the land area is little driven COVID-19 MR, but it reversely works on South and West Texas. That indicates spatial distancing is more available for South and West Texas than northern Texas. Factor 4 is adult population index the coefficient is moved from range -0.31 to −0.29 to range −0.14 to −0.1. A negative relationship with MR indicates lower mortality in younger population (but also higher mortality in elderly population). This negative association was the strongest in South and West TX but weakest in the northern TX. Factor 5 is the medical supply index with coefficient from range −0.04 to −0.03 to range 0.21–0.24. Higher BPC was supposed to have lower MR in general. Nevertheless, there were only very few TX counties with slightly negative coefficients, but most in positive. This indicates that by the fourth quarter, MR still went up despite higher BPC.

In the first quarter of 2021, Factor 1 with coefficient from range −2.14 to −1.73 to range −0.15 to −0.04 is negative related to deaths all across TX based on negative coefficients. Factor 2 becomes positive precipitation and negative land area, and it is negatively related to death across TX due to negative coefficients. That means the higher the precipitation or less land area, the less death. This is a bit counter-intuitive. Factor 3 whose coefficient is from range −0.52 to −0.24 to range 0.3–0.45 is an environmental factor of positive temperature and AQI. A positive relationship death means the higher temp and the poorer air quality caused more death, or colder temperature/better AQI caused less death. A negative relationship is the opposite. It is negative in Central to West TX, but positive in the eastern TX. Factor 4 is the adult population. It is all negative in the western TX but positive in the South TX. Factor 5 is the poor economic condition. The positive relationship indicates that the poor economic condition is affecting the West, Southeast, and the Central TX. 

#### 3.7.2. Temporal Change of CC Factors

Population and hospitalization impact on COVID-19 within the three quarters is relatively negative. For coefficients, the value of the coefficient is fixed between −2.14 and −0.04. For the movement of spatial impacts, the spatial distribution of COVID-19 impacts is stagnant across three quarters. Due to negative impacts in entire Texas population, hospitalization is not determinant of curbing Texas COVID-19 CC spread. Hence, community containment measures are the crucial result of cluster spreading as one of the characteristics of COVID deterioration.

Adult population impacts are quite a few negative in two quarters of 2020 and positive impact of 2021 the first quarter in terms of two aspects. First, the coefficients from the third quarter to the fourth quarter still account for −0.74 to 0.17. That means policy restrictions are gradually working and the virus is spreading along with spatial cluster. Second, air quality impacts during three quarters are flexible in terms of two aspects. First, the coefficient range in the third quarter increased from −0.58 to 0.38 until no exhibition in the fourth quarter. It demonstrated that the role of environment is decreasing. Second, both the areas of positive impacts with red colors and the areas of negative impacts with blue colors are moved from Northwest to Southeast Texas, from north-central to south-central, respectively. Interestingly, positive air quality impacts are shown in the first quarter of 2021. It implies that environmental impacts are still working and accelerate COVID-19 spreading.

Economic impacts during three quarters are remarkable but are the most important factors among the five factors. On the one hand, the coefficient range in the three quarters increased gradually from −0.63 to 0.48 to 0.18 to 0.77. It demonstrated that the role of economic impacts is rising with COVID-19 case growth. Second, the areas of positive impacts with red colors are increasing around East Texas, whereas the areas of negative impacts with blue colors are extending around West Texas.

Natural supply impacts in three quarters have fluctuated. First, the coefficient range within the three quarters changed from −0.52–0.24 to −0.19–0.04. It demonstrated that the role of natural supply is barely noticed. Second, natural supply has few impacts on COVID-19 expansions.

Medical supply impacts in three quarters have fluctuated as well. First, it is noticed that there was no representation of medical supply index in the third quarter, and it triggered in the fourth quarter. Its coefficient range changed from −0.04–0.03 to 0.21–0.24. It demonstrated that the role of medical supply impacts is increasing and out of control. Second, the cluster of positive impacts with red colors is in East Texas. Notably, medical supply impacts are the temporary results from no emerging in the first quarter of 2021.

## 4. Discussion

**Figure 7:**
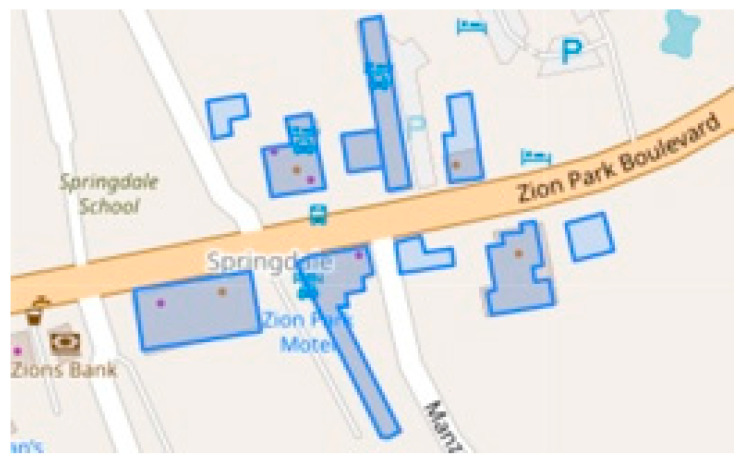
Tracing map of pioneer lodge motel.

COVID-19 virus runs rampant in high-density housing sites such as nursing homes. Emergent cluster detection is a precise way of tracking the virus. In this study, we explored three types of clustering the analysis methods. A space-time cluster’s detection of COVID-19 mortality rate is built on Kulldorff’s scan statistic method, which is the most popular in the epidemiology application. What we did first is to test the null hypothesis H0 (constant probability for all areas) and the alternative hypotheses H1(the specific area z has a larger probability than outside areas) using a Poisson model. Then we calculated the maximum likelihood and *p*-value, based on a given region z. Two clusters were pointed out that the sensitive period was July–September and November 2020–February 2021, referring to 199 counties. To narrow the tracking area, we used EM and HC clustering to further seek much better clusters. EM algorithm assists in finding out seven smaller clusters in the last quarter. HC clustering analysis directly pinpointed eight counties as a significant cluster. In fact, if the COVID-19 case data were available at street or neighborhood level, meaning the address of individual death could be better captured, specific hotspot of neighborhood or even building could be identified via GIS. HC and EM clustering provide richer descriptions of clustering structures than traditional cluster detections. Importantly, they facilitate the realization of tracing the trajectory of individual cases based on reality. For example, there is a death case at Pioneer Lodge Motel in Zion National Park in Hays county in Texas. We use ST_Buffer to build a 100-m quarantine area around the building of Pioneer Lodge in PgAdmin software (pgAdmin Development Team, California, CA, USA) in Figure 7. Next, the intersection area is selected around Pioneer Lodge Motel in Zion National Park. Finally, it is easy to use ST_Area command to find out 10 of the biggest building at the intersection area. The blue squares are identified as suspected buildings with high-density connections. Due to confidential COVID-19 patient information, our research does not incorporate patient addresses. The figure below aims to explain the possibility of the implementation of tracking the virus based on geographical cluster detection.

The purpose of GWR modeling is to find out related COVID-19 factors. That is not only because the source of COVID-19 is still a puzzle, but also because there may be a causality hidden within the correlation. In the GWR model, COVID-19 mortality rate analysis, the research period is locked at the last two quarters in 2020 according to the previous clustering analysis. We examined the inclusion of race, temperature, air quality, precipitation, hospitalization, age structure 14 variables. Furthermore, the principal component analysis (PCA) has integrated five factors related to mortality rate, including total population and hospitalization, medical supply, age structure, air quality, and economic condition. Explanatory variables are highly significant to the corresponding factors as well as in Table 6. Lastly, by defining a weight as the variance proportions for each variable, the GWR model disclosures sensitive factors in spatial-temporal variability of COVID-19 mortality rates in response to social-economic and environmental impacts in Texas counties. AQI, economic condition, and adult population indexes are regarded as sensitive factors.

Since time series are too short to be enough considered, spatial-temporal cluster detection, EM and HC clustering detection, and GWR modeling were explored to examine the imbalanced distribution of COVID-19 MR and the complex relationship with its risk factors [57]. The longitudinal monitor mechanism filled the gap of geographical analysis of COVID-19. This study has conducted some spatiotemporal analysis that provides unique insights about COVID-19, which is defined by the positions of objects within the environment, the use of dynamic time intervals, ontology or the study of the relationships of the objects, real-time or real-world modeling, and the use of analytical tools. It is a mix of conventional Geographical Information Systems (GIS) with the use of modeling and simulation skills [58].

The sensitive area is different in clustering analysis and GWR modeling due to different distribution. In cluster analysis, the sensitive areas are located at Cottle, Stonewall, Bexar, Tarrant, Dallas, Harris, Jim hogg, and Real eight counties, corresponding to Southeast Texas. Their distinction is from different mathematical distributions. Clustering methods are used by Poisson regression analysis while Gaussian distribution is applied in GRW modeling. In spatial epidemiology, mortality using a Poisson process is more appropriate than a linear scale, which the GWR is. Specifically, the Poisson regression identifies the relative risk of mortality linked with a given exposure that can represent a risk rise with some percent. Thus, clustering detection is more accurate than GWR in the forecast of mortality region [59].

Referring back to the current study, the first strength is that its performance geographically targeted ways to blunt the spread of COVID-19 as quickly as possible and save lives. Through the comparison between objective clustering techniques and traditional space-time cluster detection, we achieve an improved cluster solution. HC algorithm clustering method tracked one cluster with eight counties in the last quarter and one cluster with four counties in the third quarter, EM clustering analysis captured seven clusters in the last quarter and no cluster in the third quarter, instead of two large-scale clusters in the space-time cluster methods. The second strength is the possibility of modeling GWR on the PCA outcomes, which improved the robustness of findings based on OLS results. Furthermore, the combination of clustering analysis and PostSQL application can provide instant information that helps decision-makers and public health professionals to take immediate action to inhibit current disease spread and to save lives in the future. In addition, quick position determination can blunt the avenue of the virus spreading and save resources (time and lives).

## 5. Conclusions

### 5.1. Limitations

This research just focuses on the Texas Covid-19 scenario, the application of research cannot extrapolate to other states. We did not capture chronic disease data to support this research. As explanatory variables, they should be incorporated in future studies, although are excited to see clinical characteristics [60] and cardiovascular conditions impacts on COVID-19 health outcomes [61]. Collecting data of multiple dimensions might improve and enrich spatial variability findings of COVID-19. This research focused on spatial-temporal quarterly GWR models, yet there is a distance to be reached for daily dynamic GWR models. GTWR or more effective spatial-temporal models should be further researched in the future. COVID-19 virus spreading relies on intangible person’s mobility and social activities [62]. Due to dynamic and complicated people’s behavior, this research is fragmentation in the constantly dynamic mobility, and traced people’s trajectory with stationary geographical location. Clustering analysis is not only limited to the geographical field but also should reach to other fields such as biological subjects. For instance, a multiple sequence alignment is explored by clustering analysis, rather than using clustalW2 tools, which aims at DNA or protein multiple sequence alignment program for proteins [63].

### 5.2. Implications 

The COVID-19 pandemic revealed systemic flaws in the health distribution system and American multiculturalism. It also exposed the weakness of conservative liberalism in the US, which is hard to unify ideology in social crisis and flourish in a consistent manner [64]. This research will benefit geographical health divides evenly and provide medical service references transparently. Inspired by [58,65], who applied and compared the performance of multiscale GWR models across the United States for incident rates and death rates to account for the spatial variability of COVID-19, spatial-temporal GWR models are considered to compare the global OLS model to disclose different change of COVID-19 cumulative case in response to social-economic and environmental variables at county-level in Texas. To add spatial-temporal variability understanding of empirical COVID-19 analysis, the GWR modeling was considered on space-time detection of an emerging cluster of COVID-19 MR. Therefore, the result of this study provides new empirical evidence to support future geographic modeling of the diseases.

Space-time cluster detection, HC&EM clustering analysis, and spatial-temporal geographical weighted regression modeling of COVID-19 are crucial to improve the surveillance health system and enhancing recognition of emergency preparedness plans for local hospital. They are beneficial for the government of Texas and CDC to make appropriate scientific judgments, target vulnerable communities, distribute health care, improve disease surveillance and response systems [66,67]. Notwithstanding, COVID-19 is like a justice scale to measure each country’s execution, COVID-19 vaccine is the best way to eliminate COVID-19 death.

## Figures and Tables

**Table 1:** A-list of variables used for geostatistical analysis.

Variable Category	Variable Name	Acronym	Variable Description
Economic	Annual income	PCI	Median Household Income
	Unemployment	UEM	Percent of residents who don’t have job
Environmental	Precipitation	PCN	Mean precipitation per month
	Temperature	TPE	Mean temperature per month
	PM2.5	PM2.5	Mean PM2.5 per day
	Air quality	AQI	Mean air quality per day
	Land Area	LA	Total land area per county
Demographic	Population density	POD	Population density
	Total population	TP	Total population
	Male population	PMP	Percent of residents who are male
	Black population	PBP	Percent of residents who are black
	Population between 20–59	P59	Percent of residents who are between 20–59
	Population beyond 80	P80	Percent of residents who are beyond 80
Health	Total hospital beds	THB	Total hospital beds
	Beds per capital	BPC	Incidents per 1000 residents
Covid-19	Fatalities	TF	Total death number
	Mortality Rate	MR	Percent of fatalities case on total case

**Table 2:** Cluster comparison table.

Items	Cluster 1	Cluster 2
Time frame	6 November 2020 to 5 February 2021	6 July 2020 to 5 September 2020
Population	13,085,347	26,217,888
Neighborhood	172 counties	27 counties
Log-likelihood Ratios	4084.27	3072.54
Number of cases	12,761	3635
Expected cases	5147.24	695.01
Observed/expected	2.48	5.23
Relative risk	3.08	5.61
*p*-value	<0.0001	<0.0001

**Table 3:** The EM clustering and HC clustering analysis.

Cluster	EM (Classes to Cluster Evaluation)				HC (Classes to Cluster Evaluation)			
	Quarter 3		Quarter 4		Quarter 3		Quarter 4	
	County NO.	*p*-Value	County NO.	*p*-Value	County NO.	Probability	County NO.	Probability
0	11	0.36	10	0.27	4	55.01	8	62.78
1	11	0.1	8	0.14		4.15		3.1
2	10	0.1	8	0.07				
3	16	0.09	7	0.3				
4	4	0.09	6	0.03				
5	16	0.1	9	0.03				
6	8	0.07	4	0.02				
7	12	0.11	7	0.01				
8			6	0.04				
9			5	0.04				
10			8	0.04				
Log likelihood		−86.34		−73.25				
Incorrectly Clustered instance					251	98.04%	247	96.48%

**Table 4:** Correlation list.

Explanatory Variables	Quarter 3 Coe./Sig.	Quarter 4 Coe./Sig.
TPE	−0.265/0.000 **	−2.11/0.001 **
PCN	−0.251/0.000 **	−0.166/0.008 **
AQI	−0.121/0.054	−0.062/0.325
THB	−0.145/0.020 *	−0.176/0.005 **
BPC	−0.007/0.908	−0.018/0.781
POD	−0.203/0.001 **	−0.247/0.000 **
LA	−0.074/0.241	−0.092/0.146
PCI	0.147/0.019 *	−0.111/0.078 **
TP	−0.176/0.005 **	−0.215/0.001 **
PBP	−0.191/0.002 **	−0.082/0.194
UEM	−0.106/0.093	−0.046/0.471
PMP	0.011/0.857	0.020/0.746
P59	−0.300/0.000 **	−0.250/0.000 **
P80	0.243/0.000 **	0.183/0.00 3**

**. Correlation is significant at the 0.01 level (2-tailed). *. Correlation is significant at the 0.05 level (2-tailed). Coe. = regression coefficients; Sig. = significance level.

**Table 5:** Varimax with Kaiser Normalization Rotated principal component analysis with six iterations.

Items	The Third Quarter Component in 2020						The Fourth Quarter Component in 2020						The first Quarter Component in 2021					
	Extr.	1	2	3	4	5	Extr.	1	2	3	4	5	Extr.	1	2	3	4	5
TPE	0.80	0.14	−0.06	0.48	0.32	0.66	0.62	0.15	0.55	0.21	0.03	−0.50	0.83	0.15	0.11	0.82	0.01	0.35
PCN	0.81	0.12	−0.12	0.65	0.37	0.48	0.77	0.10	0.37	0.77	0.06	−0.16	0.78	0.13	0.85	0.06	−0.08	0.19
AQI	0.63	0.29	0.02	−0.19	0.03	0.71	0.50	0.18	0.55	0.24	−0.02	−0.33	0.83	0.05	−0.31	0.85	−0.02	0.08
THB	0.95	0.97	0.06	0.01	−0.02	−0.01	0.96	0.97	0.01	0.02	0.08	0.04	0.96	0.98	0.02	−0.02	0.06	−0.02
BPC	0.34	0.15	0.04	0.05	0.08	−0.56	0.64	0.12	0.00	0.09	−0.05	0.79	0.33	0.11	−0.08	−0.54	0.08	0.13
POD	0.93	0.95	0.12	0.10	−0.06	0.07	0.93	0.94	−0.01	0.12	0.15	−0.04	0.93	0.94	0.13	0.03	0.12	−0.06
LA	0.71	0.07	0.06	−0.80	0.18	0.15	0.61	0.08	0.20	−0.75	0.10	0.03	0.67	0.15	−0.74	0.07	0.06	0.20
PCI	0.69	0.14	0.05	0.10	−0.81	0.09	0.63	0.18	−0.73	0.09	0.07	−0.23	0.60	0.09	0.03	0.00	0.06	−0.80
TP	0.97	0.98	0.08	0.02	−0.03	0.06	0.97	0.98	0.01	0.03	0.11	−0.02	0.97	0.98	0.03	0.02	0.09	−0.03
PBP	0.59	0.29	0.27	0.51	0.31	−0.26	0.71	0.23	0.26	0.70	0.23	0.22	0.69	0.26	0.65	−0.18	0.25	0.31
UEM	0.68	0.03	0.00	0.13	0.80	0.14	0.66	0.00	0.81	0.07	0.01	−0.06	0.68	0.03	0.12	0.20	0.01	0.79
PMP	0.36	−0.14	0.53	−0.16	0.01	−0.17	0.45	−0.16	−0.02	−0.08	0.46	0.46	0.39	−0.16	−0.16	−0.23	0.54	0.04
P59	0.79	0.19	0.84	0.20	−0.10	0.08	0.78	0.17	−0.08	0.17	0.84	−0.03	0.79	0.18	0.18	0.09	0.84	−0.12
P80	0.65	−0.21	−0.77	0.05	−0.03	−0.02	0.69	−0.19	−0.03	0.05	−0.81	0.03	0.64	−0.21	0.03	0.01	−0.77	−0.02

**Table 6:** The relationship between factors and explanatory variables.

Study Period	Population and Hospitalization	Adult Population	Land Area	Economical Condition	Air Quality and Medical Care
2020 Quarter 3	Factor 1	Factor 2	Factor 3	Factor 4	Factor 5
2020 Quarter 4	Factor 1	Factor 4	Factor 3	Factor 2	Factor 5
2021 Quarter1	Factor 1	Factor 4	Factor 2	Factor 5	Factor 3
Explanatory Variables	Cor./Sig.	Cor./Sig.	Cor./Sig.	Cor./Sig.	Cor./Sig.
THB	0.97/0.00				
POD	0.95/0.00				
TP	0.98/0.00				
PCN					
PBP					
P59		0.84/0.00			
P80		−0.77/0.00			
TPE					
AQI					0.71/0.00
PCI				−0.81/0.00	
UEM				0.801/0.00	
BPC					0.78/0.00
LA			−0.81/0.00		

**Table 7:** GWR and OLS models’ comparison.

Item	The Third Quarter of 2020		The Fourth Quarter of 2020		The First Quarter of 2021	
	OLS	GWR	OLS	GWR	OLS	GWR
AICc	875.23	851.54	665.44	653.85	875.2	851.54
R^2^	0.17	0.37	0.10	0.20	0.16	0.37
Std. Deviation	0.59	0.74	0.29	0.35	0.59	0.74
Neighbors	254	128	254	201	254	128
Max_Value	−1.52	−0.57	−2.78	−2.93	−1.52	−0.57
Min_Value	−5.22	−4.92	−5.66	−4.97	−5.23	−4.92
Average	−3.18	−3.14	−2.78	−3.80	−3.18	−3.14

AICc means Akaike information criterion.

## Data Availability

All data, models, and code generated or used during the study appear in the submitted article.
